# On the interface between linguistics, computer science and psychiatry: analyzing textual key-factors affecting BERT-based classification of schizophrenia in social media texts

**DOI:** 10.3389/frai.2026.1781552

**Published:** 2026-04-08

**Authors:** J. V. Miranda e Silva, C. Rodrigues, E. Vital Brazil

**Affiliations:** 1 Programa de Pós-Graduação em Estudos da Linguagem, PUC-Rio, Rio de Janeiro, Brazil; 2 IMPA Tech, Rio de Janeiro, Brazil; 3 PUC-Behring Institute for Artificial Intelligence, Rio de Janeiro, Brazil

**Keywords:** data filtering, discussion topics, language, Natural Language Processing, schizophrenia, text length

## Abstract

**Introduction:**

This paper investigates language impairments in schizophrenia (SZ) by analyzing the decision-making process of a transformer-based model in discriminating between texts produced by persons with SZ and persons without SZ. By doing so, we integrate insights from language-centered investigations with computational approaches. Using BERT-base-cased, we explore how linguistic markers of SZ can be identified through Natural Language Processing (NLP) techniques, with emphasis on improving performance reliability via dataset refinement and approaching interpretability of deep learning outputs via statistical analyses of thematic content.

**Methods:**

We report the fine-tuning of a BERT model for text classification of 31,278 Reddit posts (15,639 SZ, 15,639 controls). The experiment evaluated the capacity of the model to distinguish language produced by individuals with SZ.

**Results:**

The model achieved moderate performance (Accuracy = 0.6969; AUC = 0.78) and remained stable across hyperparameter configurations, indicating that foundation models such as BERT fit to data and, therefore, further performance gains are more likely to be derived from dataset refinement than from additional hyperparameter optimization. There were three key factors affecting the model’s performance: text length, topic of discussion and vocabulary choices. Posts that were correctly classified tended to be significantly longer (*p* < 0.001, M = 37.30), focused on certain specific topics (e.g., r/Christianity), and contained more words related to mental health conditions, particularly those semantically related to SZ.

**Discussion:**

These factors have also been reported in manual analyses of the impacts of SZ on language. These findings contribute to the accuracy of computational models aimed at working on linguistic classification tasks and underscore the value of carefully curated datasets, while demonstrating the viability of NLP methods in profiling SZ language.

## 1 Introduction

Schizophrenia (SZ) is characterized by a spectrum of symptoms typically categorized as positive (e.g., hallucinations, delusions, disorganized speech), negative (e.g., alogia, anhedonia, affective flattening), and disorganized (e.g., deficits in executive function, memory, and verbal fluency) (APA, 2013). These symptoms broadly affect cognition, manifesting with varying degrees of severity and diverse combinations, which contribute to the complexity of SZ diagnosis. Importantly, linguistic anomalies emerge as some of the most direct manifestations of the disorder’s underlying cognitive disruptions. Linguistic research over the past decades has shown that SZ correlates with a high frequency of linguistic anomalies. Speakers with SZ are frequently reported as exhibiting linguistic deficits that can be attributed to impairments at different components of grammar. At the syntactic level, the literature has reported reduced sentential and nominal structures, as well as problems with grammatical operations on dislocation and deletion of constituents (Morice and McNicol, 1986; Docherty et al., 1996; Moro et al., 2015; Çokal et al., 2018; Chaves, 2022). Issues with combinatorial morphology (e.g., regular past tense formation) have been reported as well (Walenski et al., 2010). Semantics and pragmatics seem similarly affected: persons with SZ exhibit consistent difficulties in building linguistic reference and contextualizing speech. It is observed production of a significantly greater number of referential anomalies (e.g., vague or ambiguous use of pronouns and definite nominal expressions) and comprehension mistakes (Çokal et al., 2018, 2019; Tovar Torres et al., 2019). These findings support theories that interpret cognitive SZ-related deficits as linguistic disruptions, resulting from malfunctions of the combinatorial system underlying language (Hinzen, 2017).

The majority of the reported findings result from studies in clinical settings, with controlled data collection methodologies, typically using experimental acceptability judgement tasks or elicitation of standardized interviews or narratives, which are aimed at ensuring consistency across speakers and allowing for reliable comparisons between SZ and controls (NSZ) (Çokal et al., 2018). Notwithstanding their achievements, most of these studies present a sample-size problem, being often based on a small amount of data, which reflects hardship in recruiting participants. In this context, the advent of computational methods, particularly Natural Language Processing (NLP) techniques come in handy. NLP techniques have enabled large-scale analyses of SZ-related speech patterns across different linguistic domains, offering quantifiable measures of linguistic impairments (e.g., Cambria and White, 2014; Birnbaum et al., 2017; Rezaii et al., 2019, among others). Studies leveraging language quantification methodologies have reported successful performances at classifying SZ through language use via systematic deviations from neurotypical speech. These reports are aligned with the SZ linguistic phenomena reported by investigations that apply specialized human annotated schemes [see for example Ziv et al. (2022), Guerra (2023)]. Most recently NLP approaches to SZ have expanded, increasingly incorporating social media sources due to the vast volume of linguistic samples available on such platforms (McManus et al., 2015; Birnbaum et al., 2017; Guerra, 2023).

In sum, while clinical data remains the gold standard, social media datasets provide the amount of data necessary for training large language models (LLMs), which are currently the state-of-the-art architecture for NLP tasks. This research field faces a limitation, however: despite its advantages in volume, social media datasets present challenges related to reliability and interpretability. The literature has shown, for instance, that classification models easily rely on lexical cues for classification (Guerra, 2023). Furthermore, although LLMs achieve state-of-the-art performance in NLP tasks and excel when fine-tuned on large datasets, general understanding of their underlying decision-making processes is still limited, reducing their applicability in clinical research (Carvalho et al., 2019; Bommasani et al., 2022).

Therefore, there is still a fundamental gap: lack of empirical understanding of which textual factors most strongly influence transformer-based classifications of SZ. That is, to what extent these models rely on linguistic structures, lexical cues, or topic patterns. Most studies emphasize performance metrics, but few systematically analyze the model’s decision-making process or evaluate how dataset curation shapes interpretability and reliability, limiting contributions of NLP to theoretical linguistics and psychiatry. Against this backdrop, the current investigation conducts an exploratory analysis of key textual factors that influence a BERT-based classification of SZ and NSZ (non-SZ, control) posts from Reddit. In conducting this research, we seek to: (i) fine-tune a transformer model for SZ classification with a carefully curated dataset designed to minimize lexical and topic bias, (ii) extract data-intrinsic information (text length, SZ-related lexical content, and discussion topic) that can influence data collection and processing in SZ-language studies.

Our findings show that a state-of-the-art transformer-based model can distinguish between SZ and NSZ posts at above-chance levels (~50%). However, the analysis indicates that the model relies on three data-intrinsic properties: text length, topic of discussion (subreddit), and vocabulary choices. Posts that were correctly classified tended to be significantly longer (*p* < 0.001, M = 37.30), on certain discussion topics, and contained more or less words related to mental health conditions, with more use of words semantically related to SZ triggering SZ classifications. This highlights the central role of dataset curation in ensuring the reliability and interpretability of computational models in SZ classification.

The paper is structured as follows. Section 2 discusses, in a more detailed way, the main contributions and limitations of computational approaches to the study of SZ-related linguistic phenomena. Sections 3–5 detail our empirical study, describing the data collection and statistical methodology, reporting the obtained results, and discussing their implications to our understanding of how SZ affects language.

## 2 Schizophrenia and Natural Language Processing

In SZ studies, computational methods have been used to automate the identification of linguistic anomalies through the modeling of the linguistic patterns and idiosyncrasies outlined in section 1, providing quantifiable measures of language deficits via large-scale investigations that surpass the capabilities of traditional linguistic analysis (Birnbaum et al., 2017; Rezaii et al., 2019; Bae et al., 2021; Tang et al., 2021; Ziv et al., 2022; Guerra, 2023). The results reported in the literature are promising, documenting linguistic patterns consistent with earlier clinical and theoretical analyses.

Altogether, these results indicate that clinical/manual and automated analyses of language in SZ could work hand in hand being used in different scientific settings: manual analyses are better suited for small, controlled datasets, while computational approaches are more effective for processing large, less controlled datasets, overcoming human limitations of scale and time that often restrict large-scale linguistic research. Also, while manual analyses provide a fine-grained in-depth description of the deficits observed in SZ, computational models could offer coarse-grained overview of these deficits, orienting, thus, manual analysis.

However, it’s important to point out that, to achieve a desirable state of collaboration, automated analyses should work on the reliability of their results, considering the quality and intrinsic aspects of analyzed data. Evidence suggests that machine-learning models trained on social media data may rely heavily on surface-level cues. For instance, according to Kayi et al. (2017), when classifying social media text, an algorithm can rely more on lexical choices (e.g., words indicating negative sentiment), while McManus et al. (2015) found that words indirectly associated with psychosis were disproportionately weighted by models.

Also, while machine learning algorithms trained on linguistic features have reported high classification accuracies for distinguishing SZ from control groups, typically ranging between 80 and 90% accuracy rate (Birnbaum et al., 2017; Bae et al., 2021; Ziv et al., 2022), computer models tend to overfit to strong contextual lexical cues. This concern is addressed by Guerra (2023), who attempted to reduce lexical bias by removing posts from r/schizophrenia and imposing a five-word minimum length, still obtaining an *F*-score above 0.90. However, the r/schizophrenia subreddit is only one of many forums where discussions frequently revolve around mental health. In addition, short textual content is insufficient for analyzing structural linguistic properties, limiting the interpretability of such results (Andreasen, 1979a,b; Chaves, 2022).

There are, however, underlying aspects of language that can enhance or minimize linguistic cues for SZ. The concreteness or abstractness of a topic of conversation/narrative seems to be a case in point. Mota et al. (2014) presents a speech graph analysis of waking and dream reports orally produced by speakers of Brazilian Portuguese, indicating that narratives produced by persons diagnosed with SZ are semantically less cohesive. This lack of cohesion stands out more in dream reports. In a similar vein, Pugh et al. (2024) shows that LLaMA 3 performed better when classifying fictional narratives.

These issues exemplify the so-called “black box” concern (Bommasani et al., 2022), which refers to the difficulty of interpreting how deep learning models process information through multiple complex layers of representation. These internal transformations often yield outputs that, while accurate, lack transparency regarding underlying decision-making mechanisms. Consequently, it’s often non-trivial to identify which linguistic features are being more influential to a deep learning model’s prediction. Since deep learning architectures are currently state-of-the-art in NLP approaches, it benefits the investigation of language in SZ to make use of such architectures to identify linguistic patterns related to the disorder. However, the interpretability of the relation between input features and output classifications is important for research fields such as psychiatry and linguistics, which are concerned with clear clues that can further expand the understanding of the relation between the disorder and its symptoms, ultimately leading to a possible improvement of diagnostic methods through language. For instance, the reliance of these models on lexical SZ-related cues, as reported in several of the aforementioned studies, is less generalizable than grammatical structure reported in theoretical literature and therefore less effective for both diagnostic applications.

Therefore, it’s important to evaluate how reliably LLMs assess language in SZ, the extent to which their predictions rely on potentially biased lexical cues, and the role that textual length plays in predictions, which can allow us to further determine whether LLMs capture patterns beyond the scope of formal analyses, that typically benefit from larger volumes of linguistic content, by identifying meaningful linguistic patterns of SZ within small text samples, or whether they behave similarly to human annotators under conventional linguistic frameworks, resulting in a significant reliance on the contextual support stemming from a greater volume of linguistic content. Provoked by these concerns, and aiming at contributing to the dialogue between psychiatry, computer science and linguistics, we conducted an exploratory study to verify the performance stability of a BERT-based model on a classification task of SZ and whether or not text-length, lexical bias and topic of discussion affect the model’s predictions.

## 3 Materials and methods

### 3.1 Data collection process description

The dataset was collected from Reddit, comprising posts and comments from SZ and control users. Since mental health data are sensitive information, all samples were anonymized. Reddit was chosen due to its API accessibility and organized community structure, which facilitate large-scale data collection and analysis. The Reddit API allows researchers to efficiently retrieve posts, comments, and user metadata across various subreddits, organized as discussion topics, making it suitable for studies that can benefit from pre-established topical organization. In this context, the use of flairs provides an additional tool for researchers trying to identify and sort populations on Reddit. Flairs are customizable tags that users or moderators can attach to posts or user profiles within a subreddit. They serve as self-identifiers or thematic labels, indicating, for instance, a user’s affiliation to the theme of a specific subreddit. Thus, these custom tags, in addition to the platform’s API and abundance of available data commonly attributed to social media platforms, make Reddit an effective platform for data scraping, as already stated by the literature (Guerra, 2023).

Users composing the SZ group were identified through participation in r/schizophrenia and self-identification via flairs, which are customizable tags that users can select to represent their profile within a subreddit (e.g., “schizophrenic”). Self-identification was further validated through regular expression (regex) searches for explicit self-declarations (e.g., “I am schizophrenic”), followed by manual verification. Additional filtering was applied to exclude users with conflicting self-reported mental disorders and non-English speakers using regex searches. A final filtering step removed subreddit-specific biases, excluding those focused on mental health (e.g., r/TalkTherapy, r/MentalHealthBabies) and non-English topics (e.g., r/askfrance, r/learndutch). A single mental health subreddit (r/AskDocs) was left in our dataset to represent SZ-related content for further investigation. Posts with fewer than five words were removed, and text normalization was applied using Python’s unicodedata library (NFKD normalization, ASCII encoding). Links were removed. Stop words and punctuation were preserved. Duplicates were excluded. Control (NSZ) users were selected based on the top 10 subreddits with the highest post counts in the SZ group after all filtering was applied. We retrieved up to 100 unique users per subreddit using PRAW’s top post queries with no overlap with SZ users. The same filtering criteria (regex searches for conflicting disorders, non-English speakers, and SZ declarations) was applied to NSZ data collection, including encoding normalization, and text-length thresholding. All posts from the NSZ group were derived from subreddits that are also present in the SZ set of posts. This was performed as a way of achieving discussion topics consistency across groups.

To mitigate subreddit distribution imbalance and reduce noise, another screening was conducted excluding subreddits with fewer than 10 unique contributors. NSZ group was composed by selecting posts from the same subreddits retained in the SZ group, using a one-to-one matching strategy to balance post counts between groups. When a given subreddit lacked enough NSZ posts to match its SZ counterpart, we supplemented the insufficiency with additional posts from the top 10 most represented SZ subreddits to preserve content consistency, aligning with the sufficient scale for BERT (Devlin et al., 2019).

Finally, although our data selection procedure involved multiple filtering steps, the possibility of noise inherent to social media self-declarations must be acknowledged. Users may sincerely believe they suffer from SZ without having received a formal clinical diagnosis. To mitigate this limitation, we conducted an additional manual verification step for users included in the SZ group. Following methodological practices reported in previous literature (Zomick et al., 2019), we searched for explicit self-declarations of a SZ diagnosis in users’ posting histories. Using PRAW, we retrieved posts and comments from the final selected usernames and automatically searched for occurrences of the strings “diagno” and “schizo” within the same entry. These instances were then manually reviewed to confirm explicit statements indicating a formal diagnosis.

See the Supplementary material available at the public data repository for a selected content flowchart and a dataset of samples identified as containing explicit declarations of SZ diagnosis.^1^ See Tables 1, 2 for dataset descriptions.

**Table 1:** Data summary after content balance.

Content data	Groups			
	Target (SZ)	SZ (diagnosis)	Control (NSZ)	Total sample
Number of posts	15,639	11,599	15,639	31,278
Total number of words	613,891	470,239	480,616	1,094,507
Total unique subreddits	114	114	114	114
Mean word count per post	39.25	40.54	30.73	34.99
Unique users	183	124	428	611

**Table 2:** Content summary of dataset after content balance.

Top 10 subreddits after content balance							
Target (SZ)				Control (NSZ)			
Subreddit	Posts	Mean word count	% Posts of total	Subreddit	Posts	Mean word count	% Posts of total
AskReddit	3,740	32.65	24.33	AskReddit	4,268	25.03	27.77
teenagers	670	19.56	4.36	teenagers	970	16.67	6.31
NoStupidQuestions	437	45.91	2.84	pics	564	24.42	3.67
pics	404	19.92	2.63	NoStupidQuestions	532	34.28	3.46
AskWomen	337	64.82	2.19	AskWomen	463	48.69	3.01
trees	308	29.41	2.00	memes	391	12.74	2.54
memes	302	20.11	1.96	facepalm	278	28.41	1.81
ftm	297	41.68	1.93	news	238	37.8	1.55
facepalm	262	27.88	1.70	AmItheAsshole	235	60.69	1.53
amiugly	251	30.33	1.63	unpopularopinion	222	39.79	1.44
Others	8,631	44.76	56.16	Others	7,478	34.51	48.66

The study in its totality was submitted to and approved by the PUC-Rio (Pontifical Catholic University of Rio de Janeiro) Research Ethics Committee under protocol number 78-2024, approval reference 91-2024.

### 3.2 Model hyperparameter optimization for fine-tuning

We fine-tuned a BERT-base-case model for a classification task to distinguish between SZ and NSZ posts from the dataset described above. This model was selected due to its efficient processing and its ability to preserve casing information, which can act as insightful features in social media texts (Herring, 2012). The model was fine-tuned using a supervised learning approach. The training data was split into 80% for training and 20% for testing. Fine-tuning was performed on labeled Reddit posts using a classification head on top of BERT’s final hidden states. Given BERT’s token limit of 512 per input, longer posts were truncated, while shorter ones were padded with special tokens. In total, the training dataset contained 1,174,764 non-padding tokens. The test dataset contained 283,587 non-padding tokens.

To analyze the performance consistency of the model and identify an optimal set of hyperparameters for the present classification task, we employed Optuna, an advanced hyperparameter optimization framework. Optuna utilizes a Bayesian optimization approach with Tree-structured Parzen Estimators, allowing for an efficient search over a high-dimensional space of hyperparameters (Akiba et al., 2019). In our implementation, we optimized the learning rate within a log-uniform range from 1e−5 to 5e−5 and the number of training epochs as an integer between 2 and 5. In addition to these optimized parameters, we maintained a set of immutable hyperparameters throughout the fine-tuning process, including a per-device training and evaluation batch size of 32 and a weight decay of 0.01. The model was fine-tuned using the Hugging Face Transformers library via a Python notebook, with all other hyperparameters kept at default settings.

For each trial, Optuna selected a new set of hyperparameters and evaluated their performance based on accuracy. The study spanned 15 trials.

The fine-tuning process was conducted using Google Colab Pro with an NVIDIA A100 GPU environment. The training scripts were implemented using the Hugging Face Transformers library and PyTorch. Performance metrics were extracted using the scikit-learn (sklearn) library.

### 3.3 Exploratory analyses of the model’s outputs

We conducted the following analyses:

(a) Text length and prediction accuracy: We tested whether mean text length (extracted from the total word count of posts) significantly differed in correctly predicted posts. A one-way ANOVA was conducted with text length as the dependent variable and prediction as the independent variable.
(b) SZ-related lexical bias: Posts from the r/AskDocs subreddit were manually inspected for SZ-related lexical items. This subreddit was randomly selected during the filtering procedure of mental health-related subreddits described in Section 3.1 and retained in the dataset as a control condition, allowing us to assess potential lexical bias associated with specific discussion topics. From the total sample of 18 posts derived from the training and test split described in section 3.2, those containing mentions of psychosis, SZ (e.g., schizophrenia, schizophrenic), or medications commonly prescribed for such conditions (e.g., Clozapine, Olanzapine) were tagged as presenting SZ-related lexical bias. The presence of such terms was marked regardless of contextual polarity: lexical items were annotated even when they appeared in negated statements (e.g., “I do not have schizophrenia”), as their occurrence was considered, for the purposes of this investigation, a potential source of lexical bias. Odds Ratio tests of independence assessed whether the presence of SZ-related terms was associated with correct model predictions. See below a representative post sample that includes the presence of a SZ-related lexical bias, highlighted in bold (see also Supplementary material):

“Is this normal back posture? Male, 22, Caucasian, Medical history: Diagonosed **Schizophrenic**, Medications: **Olanzopine,** 15 mg, Height 5′ 9/10″, Weight: 7 stone (I know, im really underweight). Stood straight for photo, any concerns?”

(c) Discussion topics and prediction accuracy: Chi-Squared tests of independence were applied to examine the association between subreddit membership and prediction outcomes. Subsequently, a logistic regression model was fitted using r/AskReddit (the most represented subreddit) as the reference category to test whether other subreddits were significantly associated with correct model predictions.

Analyses of (a) and (b) were conducted in JASP (version 0.19.0.0). Logistic regression modeling was implemented in Google Colab using the libraries pandas, numpy and statsmodels. Visualizations were generated using matplotlib and seaborn. Datasets and Python notebooks are publicly available in the project’s repository.

## 4 Results

### 4.1 Optuna hyperparameter study and best model’s performance

The 15 Optuna trials were assessed based on validation accuracy, with the best model achieving an accuracy of 0.7022. The table below presents the details of each trial, including training loss at different steps and final validation accuracy. Note that trials that chose a number of epochs lower than 3 did not reach 2000 training steps. After evaluating all trials, the best-performing combination of hyperparameters regarding accuracy were learning rate fixed at 1.41e-5 and number of epochs at 3. See Table 3 for results.

**Table 3:** Results of the hyperparameters Optuna study.

Trial	LR	Epochs	1st Loss	2nd Loss	3rd Loss	4th Loss	Accuracy
1	1.25e−5	3	0.6152	0.5705	0.5376	0.4775	0.7011
2	1.11e−5	3	0.6183	0.5751	0.5446	0.4933	0.7019
3	1.98e−5	3	0.6183	0.5681	0.5249	0.4278	0.6963
4	3.36e−5	2	0.6204	0.5646	0.5105	–	0.7000
5	2.93e−5	2	0.6143	0.5580	0.5068	–	0.6973
6	3.64e−5	3	0.6206	0.5631	0.5136	0.3524	0.6870
7	2.79e−5	4	0.6221	0.5713	0.5203	0.3656	0.6762
8	1.76e−5	2	0.6168	0.5714	0.5342	–	0.7006
9	3.85e−5	3	0.6216	0.5645	0.5135	0.3387	0.6947
10	2.48e−5	5	0.6188	0.5688	0.5289	0.3852	0.6845
11	1.07e−5	5	0.6169	0.5731	0.5426	0.4768	0.6893
12	1.01e−5	4	0.6175	0.5745	0.5450	0.4882	0.6931
13	1.41e−5	3	0.6153	0.5717	0.5379	0.4640	0.7022
14	1.44e−5	4	0.6168	0.5704	0.5338	0.4500	0.6971
15	1.57e−5	3	0.6153	0.5704	0.5327	0.4485	0.7006

Best hyperparameter configuration highlighted. “LR” stands for “Learning Rate,” while losses represent sets of 500 training steps.

Once the optimal hyperparameters were identified, a new model was fine-tuned from scratch using the best hyperparameter configuration. Results are presented below.

Training: Loss decreased from 0.6149 (step 500) to 0.4571 (step 2000). Evaluation: Loss = 0.5933, Accuracy = 0.697, Precision = 0.686, Recall = 0.717, F1 = 0.701. TP/FP/TN/FN = 2224/1019/2136/877; Runtime = 36.8 s.

To assess whether the model’s performance differed significantly based on the reliability of disclosed diagnoses, we manually verified posts from users who explicitly stated having a SZ diagnosis (e.g., “I am diagnosed schizophrenic” or similar phrases, as opposed to more general statements like “I have schizophrenia”), as described in section 3.1. Out of the 183 SZ users in our dataset, 124 (67.76%) explicitly disclosed diagnosis. When evaluating the model’s performance on this verified subsample (all NSZ vs. diagnosis-confirmed SZ), an accuracy of 0.6972 was observed, which was nearly identical to the performance observed on the full dataset.

**Figure 1:**
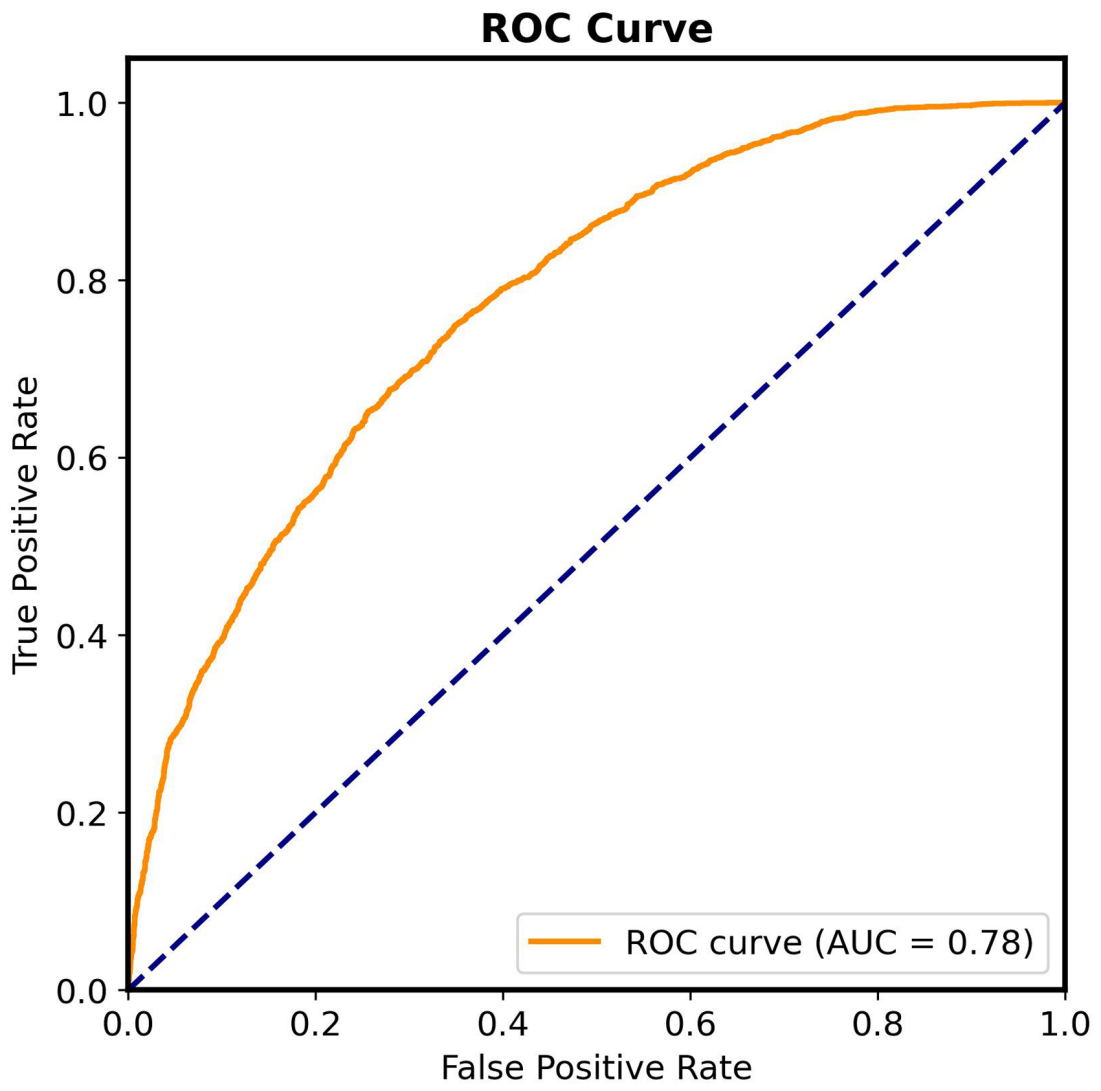
Receiver Operating Characteristic (ROC) curve for the evaluation of the fine-tuned BERT model on the SZ vs. control classification task. The curve illustrates the trade-off between true positive rate and false positive rate across classification thresholds (AUC = 0.78).

To further assess the model’s performance, a Receiver Operating Characteristic (ROC) curve is displayed (Figure 1), resulting in 0.78 AUC.

### 4.2 Impact of text length

**Figure 2:**
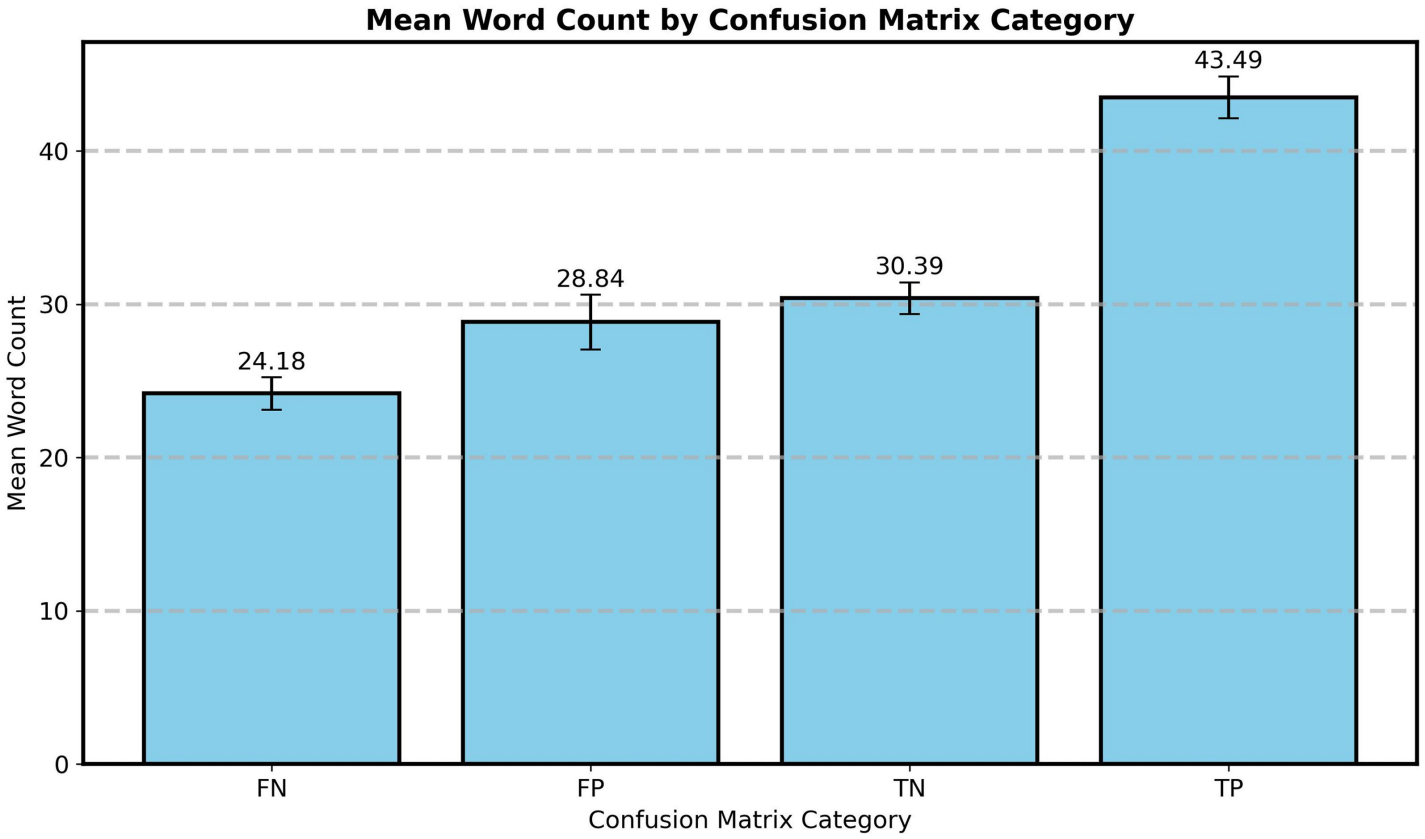
Word counts of Reddit posts by confusion matrix category (true positives, true negatives, false positives, and false negatives) with standard error bars.

Figure 2, showing the mean text length for each prediction class (TP, FP, TN, FN), indicates a notable difference between correct prediction categories (TN, TP) and incorrect ones (FN, FP), with the correct predictions exhibiting a higher mean text length.

The results of the one-way ANOVA for text length across the four evaluation categories (TP, TN, FP, FN) reveal significant differences between correct and incorrect predictions (Table 4). There were significant differences in text length across categories, with an *F*-value of 38.486 and a *p*-value of less than 0.001. *Post hoc* comparisons revealed significant differences between TP and the other categories, particularly with FN, where the mean difference was the highest (19.312). FP and FN did not show a significant difference (*p* = 0.245), so as TN and FP (*p* = 0.876). Since text length (word count) distributions were non-normal, Kruskal–Wallis tests were conducted as a non-parametric robustness check. The test showed the same significance pattern as the ANOVA test (*p* < 0.001), supporting results. The effect size for the ANOVA was small (*η*^2^ = 0.018).

**Table 4:** One-way ANOVA and *post hoc* comparisons of both the test dataset.

ANOVA comparisons						
ANOVA—text length						
Cases	Sum of squares	df	Mean square	*F*	*p*	*η* ^2^
Evaluation	339995.583	3	113331.861	38.486	**<0.001**	**0.018**
Residuals	1.841 × 10^+7^	6,252	2944.721			

*Post hoc* comparisons—evaluation					
Optuna results		Mean difference	SE	*t*	*p*tukey
TP	TN	13.106	1.644	7.972	**<0.001**
	FP	14.658	2.053	7.140	**<0.001**
	FN	19.312	2.164	8.925	**<0.001**
TN	FP	1.552	2.066	0.751	0.876
	FN	6.207	2.176	2.852	**0.023**
FP	FN	4.655	2.500	1.862	0.245

Best hyperparameter configuration highlighted.

### 4.3 Lexical-cues bias

We tested r/AskDocs, a subreddit randomly selected to represent the broader theme of health. To evaluate effects of lexical bias, we manually reviewed all 18 samples from the subreddit and tagged posts containing mentions of psychosis, mental illness, or medications commonly prescribed for such conditions (e.g., clozapine). We then conducted Odds Ratio tests of independence to examine whether the presence of SZ-related terms was associated with correct model predictions.

**Figure 3:**
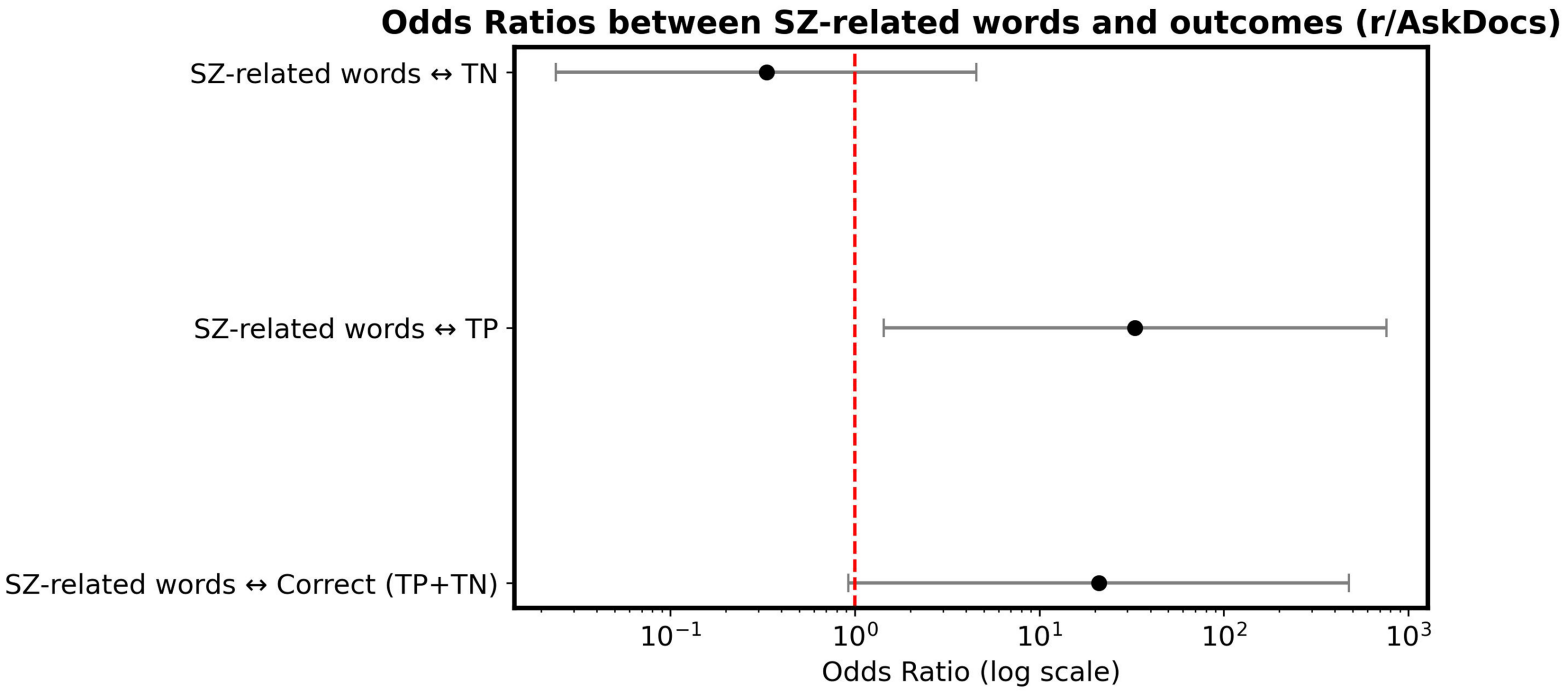
Odds ratios indicating the association between SZ-related lexical items and the classification outputs of the BERT model for posts from the r/AskDocs subreddit.

A strong association between the presence of SZ-related words and the model’s classification outcomes was found (Figure 3). Posts containing SZ-related terms were more likely to yield correct classifications (TP or TN). However, the confidence interval was wide and approached the line of no effect (red dashed line), reflecting the limited sample size and associated uncertainty. Posts mentioning SZ-related terms were significantly more likely to result in TP, representing the only association that clearly exceeded the no-effect threshold. In contrast, the Odds Ratio for TN was below 1, indicating no meaningful relationship and suggesting that such terms did not contribute to correct non-SZ predictions. More details on the Odds Ratio tests of independence and post samples tagged as containing SZ-related lexical bias are available in the supplementary materials within the public data repository.

### 4.4 Topic of discussion

In the present study, discussion topics refer directly to the subreddits in which posts were published. No additional topic modeling or semantic clustering procedure was applied. Thus, subreddit membership was used as a proxy for discussion topics, given that each subreddit is organized around a specific theme. Chi-Squared tests of independence were conducted to examine the relationship between discussion topics and classification outcomes (i.e., TP, FP, TN, FN). Results revealed a statistically significant association between subreddits and classification outcomes [*Χ*^2^(113) = 194.680, *p* < 0.001], with an effect size, measured by Cramer’s *V*, of 0.176, indicating a small to moderate association. To further explore which subreddits contribute most to correct predictions (TP, TN), we conducted an additional logistic regression analysis using subreddits as predictors. To examine whether certain subreddits were more likely to yield significant results, we set r/AskReddit as the reference category in the logistic regression model, as it was the most represented subreddit and provided a stable baseline for comparison. To address the large number of subreddits (114), we also estimated an L1-regularized logistic regression (LASSO). LASSO regularization performs variable selection by shrinking unstable coefficients. Still, all subreddits retained non-zero coefficients.

The model’s summary *p*-value of <0.001 indicates that the influence of subreddits on correct predictions is statistically significant. The Pseudo-R2 of 0.026 indicates that the model explains about 2.6% of the variance in log-likelihood compared to the null. Several subreddits were significantly associated with correct predictions, as indicated by the Wald test. Posts from r/Advice, r/AskWomen, r/Christianity, r/ftm, and r/suggestmeabook were significantly more likely to yield correct classification outcomes. In contrast, posts from r/CasualConversation, r/PublicFreakout, r/RoastMe, r/mildlyinfuriating, and r/pics were significantly less likely to result in correct predictions.

The results are summarized in Table 5, which shows the results of a logistic regression model examining the relationship between subreddit, inferred as discussion topics, and the likelihood of correct classification outcomes.

**Table 5:** Logistic regression model summary—correct outcomes (TP, TN) as a function of subreddits.

Model summary—correct outcomes (TP, TN) as a function of subreddits							
Model	Deviance	AIC	BIC	df	Δ*Χ*^2^	Pseudo *R*^2^	*p*
*M*_subreddits_	7477.6	7705.6	8475.4	6,142	197.8	0.026	<0.001

Coefficients					
Subreddit	Estimate	SE	*z*	df	*p*
Intercept^a^	0.590	0.184	3.211	1	0.001
r/Advice	0.908	0.347	2.615	1	0.009
r/AskWomen	0.464	0.198	2.339	1	0.019
r/CasualConversation	−1.095	0.424	−2.582	1	0.010
r/Christianity	1.401	0.433	3.235	1	0.001
r/PublicFreakout	−0.501	0.254	−1.976	1	0.048
r/RoastMe	−1.292	0.373	−3.467	1	0.001
r/ftm	0.820	0.368	2.229	1	0.026
r/mildlyinfuriating	−0.538	0.230	−2.340	1	0.019
r/pics	−0.388	0.150	−2.587	1	0.010
r/suggestmeabook	1.652	0.738	2.239	1	0.025

Estimates based on binomial categories (0, 1). “0” represented incorrect classifications, while “1” represented correct classifications.

a Intercept is based on r/AskReddit, while the subreddits presented on this current table are restricted to significant ones (*p* < 0.05) from the remaining 113 subreddits.

## 5 Discussion

The Optuna hyperparameter optimization yielded minimal variation across trials, with accuracy values clustering closely around 0.69–0.70. This confirms prior observations that transformer-based models fine-tune efficiently even with modest tuning and that performance differences within a narrow hyperparameter space are small when the dataset is sufficiently large (Devlin et al., 2019). The model’s final results are significant, as an accuracy near 70% and an above-chance AUC (0.78) suggest it learned meaningful linguistic distinctions to discern classes rather than relied on random guessing. The relatively balanced trade-off between Precision (0.686) and Recall (0.717) indicates that the model is not biased toward either class, but rather identifies linguistic features that reliably differentiate SZ from NSZ in correct predictions. Furthermore, the consistent performance of the BERT-based model across multiple Optuna trials suggests that the transformer architecture underlying LLMs is capable of adequately representing language in order to identify patterns that are relevant to the classification of SZ texts. Thus, future efforts may be more impactful if directed towards improving dataset quality, thus addressing the classification challenge from a different axis.

Notably, our model’s performance is lower than that reported in studies utilizing less curated datasets. For instance, Guerra (2023) reported an F1-score above 0.91 using a similar BERT-based architecture. This discrepancy underscores the importance of dataset composition: when lexical or topical cues are overlooked during training or fine-tuning, models may report high scores but rely on features that are tangential to linguistic impairments associated with SZ. By contrast, stricter filtering, as implemented here, seems to reduce this inflation, resulting in performance levels comparable to those from standardized clinical datasets (Kayi et al., 2017). This suggests that approximately 0.70 accuracy performance represents a baseline for models trained on data where lexical bias is minimized.

Our main observations supported that text length positively contributes to classification accuracy. Posts categorized as correct predictions (TP, TN) consistently exhibited higher word counts, while shorter posts yielded more misclassifications. This suggests that longer texts provide the model with greater contextual density, potentially including structural discourse markers (e.g., sentence embeddings, functional categories, etc.), more coherence relations, and even more lexical cues that facilitate prediction. However, the specific relationship between grammatical structure, coherence and the attention allocated by the model to such structures remains an open question not addressed in the present study.

These findings align with clinical-standardized linguistic research showing that SZ-related anomalies are more observable in extended discourses. Andreasen (1979a,b) showed that longer utterances increase the likelihood of observing SZ linguistic patterns, and Docherty et al. (1996, 1997) required at least 10 min of speech to capture communicative failures. In accordance, Chaves (2022) found that 30-s narratives (average 37.6 words) were insufficient to reveal SZ-specific grammatical markers. These observations are rather expected: longer discourse production naturally results in a larger amount of information, increasing, thus, statistical measures of grammatical structure (e.g., means and ratios). Therefore, longer linguistic productions tend to expose cumulative disruptions in, for instance, coherence and structural organization. For computational models, longer inputs similarly increase the number of classification cues available. Consequently, minimum-length thresholds should be treated as methodological precautions in computational studies, helping ensure that analyses are based on texts that contain sufficient linguistic material for meaningful classification.

Also, as shown in 3.3, the performance metrics of models trained or fine-tuned on less rigorously filtered datasets may be inflated due to lexical biases. Our analysis of the r/AskDocs subreddit revealed that posts containing explicit SZ-related terms were significantly more likely to yield TP predictions. Sample size was small (18) and, consequently, results should be interpreted as seminal rather than conclusive. However, this pattern mirrors concerns raised in the literature about lexical overfitting in mental-health NLP models (McManus et al., 2015; Kayi et al., 2017). Even after extensive dataset filtering, results indicate that the model still relied on explicit disorder-related vocabulary when available. This has implications on interpretability and generalizability of NLP models. Regarding the former, high performance in health-adjacent subreddits, or their overrepresentation in datasets, may reflect attention to lexical cues rather than grammatical structure. Models trained under such conditions risk misclassification in contexts where mental-health vocabulary is absent, limited, or culturally encoded differently. This impacts generalizability and is especially relevant for cross-linguistic research and for applications involving populations with varying literacy or discourse norms. Thus, we conclude that, while lexical cues are difficult to be entirely eliminated from datasets, their influence must be quantified and controlled to prevent misleading optimistic interpretations of classification performance.

One matter in question here is the fact that speakers with SZ produce more words with morphological and phonotactic errors. Walenski et al.’s (2010) study on the formation of regular and irregular past tense forms is informative on this issue, indicating that, compared to controls, speakers with SZ have significantly more difficulty in building the past tense form of regular verbs. There was no significant difference in irregular forms. Thus, considering that irregular forms are stored in the lexicon as idiosyncratic items, while regular forms are derived morphologically, Walenski et al. (2010) suggest that procedural memory is impaired in SZ, with relative preservation of lexical memory. Our results point only to the need to reduce the volume of lexical information semantically related to mental health conditions. In convergence with the literature, we emphasize the importance of preserving morphological and phonotactic errors, which are in fact grammatical markers of SZ.

The logistic regression revealed that the inclusion of subreddits as predictors significantly improved model fit (Δ*Χ*^2^ = 197.8, *p* < 0.001). Text-oriented subreddits involving suggestion- or advice-giving or personal disclosures (e.g., r/Advice, r/AskWomen, r/Christianity, r/suggestmeabook) were associated with higher rates of correct predictions, whereas subreddits associated with lower accuracy were mostly visually oriented (e.g., r/pics, r/RoastMe). Even though the literature hypothesizes that topicality may impact the saliency of semantic coherence impairments (Mota et al., 2014), it’s not yet possible to interpret the present results as evidence that BERT captures deeper grammatical or coherence disruption patterns related to topicality and SZ. Instead, topic differences likely influence the density of linguistic information available to the model (visual-oriented vs. text-heavy topics), the register or communicative style associated with different social media contexts (Herring, 2012) and the stability of discourse structure, which may vary across genres. For example, advice-oriented posts tend to include explicit temporal-causal sequences (e.g., “this happened, so I did X,” “this is the author who also wrote X”), whereas subreddits centered on images or jokes rely on short, informal replies with fewer linguistic contextual cues.^2^

This stresses that topic curation should, for now, be treated primarily as a methodological variable. Consequently, analyses that draw conclusions about SZ-related language should consider the interaction between linguistic features and discourse genre. However, one subreddit that was associated with a significantly negative impact on the model’s predictions, r/CasualConversation, was not image-centered and exhibited a mean length (~42 words) comparable to the overall mean observed across correct prediction classes. This indicates that text length and topic do not appear to interact directly in shaping prediction outcomes. Rather, each seems to contribute additively, without strong interaction. This is supported by the text-length ANOVA, which indicates that prediction classes accounted for only ~2% (*η*^2^ = 0.018) of the variance in post length. Further research is therefore needed to examine the linguistic patterns characteristic of subreddits positively associated with accurate classification and to compare them with those of r/CasualConversation. Investigating structure-oriented features (e.g., POS distributions, syntactic depth) alongside lexical patterns and/or semantic networks would help clarify which linguistic cues the model finds most informative and how these cues interact with dataset-intrinsic properties such as topic and length.

To conclude, we have shown that a BERT-based model achieves moderate success classifying posts/texts from SZ and NZS Reddit users. When it runs unconstrained it’s reported as achieving a better performance. However, as shown, it tends to use SZ-related words to do the required classification when such cues are available, impairing generalizability. Although the proportion of samples identified as exhibiting lexical bias was small (*n* = 18), we do not claim that this analysis provides statistically robust evidence. Rather, it should be interpreted as a qualitative exploratory step aimed at gaining insight into how the model may be learning linguistic patterns associated with SZ. Regardless, this finding carries implications for the model’s broader applicability. In particular, its performance may decline in contexts beyond social media, where linguistic production differs from online posts and where explicit SZ-related lexical markers may be less salient or entirely absent (e.g., in clinical interviews). Such contextual differences raise concerns about the robustness of the model’s predictions across domains. Accordingly, future research should evaluate the model’s performance on linguistic data derived from alternative settings. We hypothesize that the model will retain moderate performance across contexts, given the careful curation of its training dataset.

Also, we observed that short posts induce more classification errors, whereas extensive posts lead to classificatory precision. While this study advanced our understanding of the relationship between data-intrinsic features and the computational assessment of SZ, some limitations must be acknowledged. First, it relies on self-declared diagnoses from Reddit users, which is in itself a noise in the dataset that can cause misclassifications from outset. We tried to reduce this noise filtering the data as discussed on section 3.1, but future work should improve this methodology. Second, while we evaluated performance across multiple hyperparameter configurations, our experiments were confined to a BERT-based architecture. Future studies could leverage the fine-tuning dataset derived from our methodology to evaluate the robustness and comparative performance of other model families, which could ultimately support the importance of dataset curation.

Given the goals of our investigation, grammatical issues were not considered in this study. We acknowledge that future studies should incorporate syntactic analyses to further our understanding on how SZ affects language and on how LLMs perform classification tasks of the sort discussed here.

Overall our results indicate that LLMs perform similarly to manual analyses based on data collected in clinical settings. Both take into account macro factors such as text/narrative length, topic of discussion, and vocabulary choices. Furthermore, the influence of these factors seems independent of the language modality (oral vs. written) used as input data. They are, therefore, preponderant factors in classificatory tasks. For that reason only they ought to be highlighted.

## Data Availability

The datasets presented in this study can be found in online repositories. The names of the repository/repositories and accession number(s) can be found in the article/Supplementary material.

## Footnotes

1 The public data repository can be found at: https://doi.org/10.5281/zenodo.18745421.

2 See the Supplementary material in the public repository for examples of posts drawn from the relevant subreddits (e.g., r/pics, r/Advice).

## Author’s note

This manuscript was previously made available as a preprint at: https://doi.org/10.1590/SciELOPreprints.14646.
