# Supplementary material for: On the interface between linguistics, computer science and psychiatry: analyzing textual key-factors affecting BERT-based classification of schizophrenia in social media texts
Source: Front Artif Intell. 2026 Apr 8;9:1781552. doi: 10.3389/frai.2026.1781552 (PMC13099882; doi:10.3389/frai.2026.1781552)
Supplement: Supplementary file 1 [file Data_Sheet_1.pdf]

AskDocs Manual Tagging

label	text	pred	eval	is_ correct	SZ_ mentioned	is_ TP	is_ TN
1	Right side of leg feels like razor blades cutting and scabs 37 male 329 pounds and losing more. I was not working for 4 years and just got a job a few months ago since then my leg feels like it's being cut on the inside and I have little red bumps that are scabbing over. Even when I cough sometimes it hurts that side of the leg.	1	TP	1	0	1	0
0	My stool was pale one time but went back to normal brown color in next bowel movement. [removed]	1	FP	0	0	0	0
1	Is this normal back posture? Male, 22, Caucasian, Medical history: Diagonosed Schizophrenic, Medications: Olanzopine, 15mg, Height 5' 9/10", Weight: 7 stone (I know, im really underweight)<br><br>Stood straight for photo, any concerns?	1	TP	1	1	1	0
1	Alergies to dishwashing? [wtf](<br><br>37 male 333 lbs 6"3 I used to wash dishes in the past with no gloves and never had too much issue. But the water here is scalding so I bought latex and pvc gloves things were fine til I put on the pvc gloves one day and they started feeling like fiber glass. See attached post for pictures. Thanks for your time	0	FN	0	0	0	0
1	Can Anti-psychotics kill me? Someone posted in r/schizophrenia that Anti-psychotics can shorten life expectancy by 30 years, and that it can cause brain damage and kill you instantly at any time. Is this true??<br><br><br><br>I am almost 29 years old, female, I am 4'11 and 145 lbs. I have had Schizophrenia for the past 8 years now, it runs in my family. I do not drug or smoke. I am on Abilify 10mg and Trazadone 50mg. I want to start taking Latuda, Haldol, and Hydroxozine though when I see my new Psychiatrist next month.<br><br><br><br>I am really worried about this. I know going off my medication is a bad idea... but if they can hurt me, I	1	TP	1	1	1	0

	have to stop.						
1	Does my finger look infected? My cat scratched me hard right in the crease part of my finger and now it's really sensitive and red:<br><br><br><br>Here's about me:<br>28 years old<br>4'11<br>Overweight<br>Schizophrenic<br><br>Meds:<br>Clozapine 800mg<br>Propanolol 10 mg 2x day<br>Aristada 662<br><br>So, is the finger just in the process of healing or does it look infected?<br><br>Thanks for any help!	1	TP	1	1	1	0
1	Because I don't have Schizophrenia. I'm hearing real people from another dimension and I know it. There's nothing wrong with me and the meds are just blocking my ability to hear them as well as I used to. I'm not afraid of them anymore and I just want to save them. I might call my doctor tomorrow and insist she tells me how to get off them without any physical issues.	1	TP	1	1	1	0
0	Is it safe to take two brand of acetaminophen in the same day [removed]	1	FP	0	0	0	0
1	When is sleep apnea an emergency? [removed]	1	TP	1	0	1	0
1	Thank you so so much! I'll call them aaap!	1	TP	1	0	1	0
1	Rash all over my forehead, help! Near my scalp down to my right eyebrow, it was very dry with white flake on top of pink skin.<br><br>[Here's an image, but the image is after I applied tea tree oil to it so you cant see the white dry bits that were there before.](	1	TP	1	1	1	0

	Age - 24<br><br>Sex - female<br><br>Duration of complaint - just today<br><br>Location (Geographic and on body) - forehead<br><br>Any existing relevant medical issues (if any) - I had shingles in august of 2014, could it be that again?<br><br>Current medications (if any) - Klonopin, Clozapine, Welbutrin						
0	Would also like to know	0	TN	0	0	0	1
0	Some people respond this way to weed, its not a mental illness. I used to smoke weed and it was a totally normal high, now if I do it causes psychosis for me	0	TN	1	1	1	1
1	Do I have an eating disorder? Male, 22, Height 5' 10", Weight 44Kg / 97lbs / 7 stone (roughly) - Diagnosed with Schizophrenia, White / British. - I don't drink alcohol or smoke<br><br>In regards to having an already low weight as it is, I have quite the attitude toward eating; I like to stay lithe and have a strong mentality in staying that way. I will forcibly keep myself from eating large portions of food despite wanting to eat - Sometimes, I will only have one meal a day at most with the occasional chocolate bar or so. I have thought about telling my GP but getting recommended to a dietitian, I would feel is pointless because I have to be the one to change how I eat and would be resilient to do so to stay thin.<br><br>Does this confirm an eating disorder and/or behaviours?	1	TP	1	1	1	0
0	I get this random lump in the throat sensation that turns painful to swallow for a couple of days then goes away completely for months. (Im on mobile so please excuse if bad formatting)<br><br>Age- 26<br>Sex- F<br>Height- 57<br>Weight- 135<br>Race- Caucasian<br>Duration of complaint- 1.5 years ago	0	TN	1	0	0	1

	Location on body- Throat<br>Any medical issues- None<br>Current medication- None (B vitamins, supplements)<br><br>This started about a year and a half ago, (it was a very stressful time in my life) my throat started hurting one day like I had scratched it from swallowing something sharp. The next day it felt normal again. I chalked it up to maybe eating sharp chips or stress and/or anxiety because I was having other anxiety related symptoms such as headaches at that time.<br><br>Ever since then, that has happened with my throat about 5 more times since.<br><br>Yesterday it flared up again. It starts with a lump in the throat feeling, turns painful to swallow for a couple of days, then goes away completely and wont happen again for months.<br><br>It doesnt feel like a sore throat from a cold. It almost feels like my throat muscles are super tight and constricted. I even looked at my tonsils this morning with a flashlight and they looked fine. No redness, no swelling, nothing out of the ordinary.<br><br>Ive tried to think back on anything Ive ate or done to cause the pain. I do get occasional tonsil stones that I tried to dig out last weekend. I also felt a tonsil stone in the back of my mouth early this morning when I had just got up. I do eat chips occasionally and I was kind of worried about something yesterday. Im just curious as to what could be causing this.						
1	I don't know what to believe. I googled, "Can Clozapine cause brain damage?" because I've been on Clozapine 800mg(I think the highest dose) for like 4 or 5 years until this past month, and it says this, "Neuroleptics - such as Clozapine, Olanzapine, Risperidone and Seroquel - are the "primary treatment" for psychosis, particularly schizophrenia. ... The evidence shows, she says, that antipsychotics not only do not work long-term they also cause brain damage - a fact which is being "fatally" overlooked."<br><br>This is really scaring me. I don't want to get bad symptoms again. I've already had really bad symptoms recently, seeing things and hearing things... feeling like things are true when others say they aren't. I don't know what to believe anymore. I don't want to get brain damage from antipsychotics. I don't want to die from them. Not sure what to do here.	1	TP	1	1	1	0
1	I have vivid nightmares that keep me up if I don't take something to help me sleep. 25F, schizophrenic, previously diagnosed with PTSD. I can't take Ambien/most prescription sleep medications because of the fact that they can cause me to become delusional, so I take an OTC sleep aid. However, recently due to	1	TP	1	1	1	0

	the blizzard terrorizing the US, I have run out. It's been a problem for years, I'll fall asleep, have a nightmare that feels like it's happening to me (usually a PTSD flashback) and I'll wake up unable to go back to sleep because it still feels like it just happened.<br><br>I feel like consistently taking OTC sleeping meds isn't healthy, but I can't sleep for more than 2-3 hours without them. That and my nightmares cause me to kick, roll, and headbutt in my sleep according to my fiance, and I have rolled off of my bed and hurt myself by falling on several occasions during these nightmares. As bad as it sounds, I can't take melatonin because it isn't "strong enough" and the nightmares still happen. I usually take the "for sleep, not a cold" diphenhydramine, because while it leaves me incredibly groggy, it makes me either not dream or not feel like I'm dreaming.<br><br>Is this normal for these conditions? Is my solution the healthiest option? And what can I do to sleep while I'm snowed in?						
1	110 heart rate and chest pain I don't know if I have just had too much caffeine today(close to 400mg) or if something is wrong. My chest feels tight and I tested my heart rate with an app on my phone and it says 110.<br><br><br><br>Should I be worried or will it just go away? I just took some ativan hoping it would calm things down.<br><br>Here's some info:<br><br>* Age -27<br>* Sex - F<br>* Height - 4'11<br>* Weight - 150 lbs<br>* Duration of complaint - 2 hours or so<br>* Location (Geographic and on body) - chest<br>* Any existing relevant medical issues (if any) - Schizophrenia<br>* Current medications (if any)<br><br>Meds:<br><br>Clozapine	1	TP	1	1	1	0

	Topomax<br><br>Buspar<br><br>Propanolol<br><br>Trazadone<br><br>Ativan<br><br>Lexapro<br><br><br><br>Should I just wait for it to resolve on its own or is it something serious?						
